# Childhood overweight and obesity among the Saudi population: a case-control study among school children

**DOI:** 10.1186/s41043-021-00242-1

**Published:** 2021-04-07

**Authors:** Hanan Aljassim, Hoda Jradi

**Affiliations:** 1 King Saud Bin Abdulaziz University for Health Sciences, Riyadh, Saudi Arabia; 2 Prince Sultan Military Medical City, Health Education Administration, Riyadh, Saudi Arabia; 3 King Abdullah International Medical Research Center, Riyadh, Saudi Arabia

**Keywords:** Children, Obesity, Overweight, BMI, Saudi Arabia

## Abstract

**Background:**

Childhood obesity is a global public health concern with major consequences. In Saudi Arabia, the percentage of children who are overweight or obese has significantly increased in the past two decades, raising concerns about the physical and psychosocial consequences of this burden. This study aimed at investigating the different risk factors contributing to childhood obesity in Saudi Arabia.

**Methods:**

A case-control study was conducted among 492 school children (246 overweight/obese children, and 246 normal weight control children aged 5-9 years). Using valid and reliable instruments, parental and child characteristics, behavioral practices, screen use, and other activities were assessed as risk factors for childhood obesity using logistic regression analysis.

**Results:**

An unemployed father (OR=11.90; 95% CI: 7.47-18.93), a father with overweight/obesity (OR=2.04; 95% CI: 1.40-2.96), an incorrect parental perception of child’s weight status (OR=2.54; 95% CI: 1.75-3.68), cesarean delivery (OR=2.52; 95% CI: 1.56-4.09), daily time in active play for less than 30 min (OR=2.18; 95% CI: 1.44-3.28), frequent snacking (OR=1.74; 95% CI: 1.05-2.93), and screen time use for more than 2 h per day outside of school (OR=1.62; 95% CI: 1.12-2.34) were all independent risk factors for being overweight or obese among the selected cases.

**Conclusion:**

Efforts to prevent childhood overweight and obesity in this population should focus primarily on the early identification and confrontation of risk factors. Such risk factors include parental characteristics and awareness of the magnitude of the burden obesity poses, behavioral practices such as frequent snacking, screen time use, and physical activity.

## Background

Obesity is one of the most pressing global public health challenges of the twenty-first century. Among children in developing countries, the number of those who are overweight or obese is 30% higher than in developed countries [1]. In 2025, the number of overweight or obese infants and young children worldwide is projected to reach 70 million [2].

Being overweight or obese during childhood is known to have negative impacts on physical and mental health [3]. Obese children are usually more likely to stay obese into adulthood and to have chronic diseases such as diabetes, hypertension, cardiovascular disease, musculoskeletal disorders, and certain types of cancer (endometrial, breast, and colon) [1, 4]. Additionally, being overweight or obese affects self-esteem of children and impairs social development [3].

In Saudi Arabia, the percentage of childhood and adolescents who are overweight or obese has significantly increased in the past two decades, raising concerns about the physical and psychosocial consequences of childhood obesity [2]. In Saudi Arabia, nearly 20% of Saudi males aged 5-9 years and 24% of Saudi males aged 10-14 years are obese [4]. The prevalence of obesity among Saudi females is 40% between the ages of 5 and 9, and 41% between the ages of 10 and 14 [4].

During the last decade, Saudi Arabia has experienced a prosperous economy that has led to drastic changes in lifestyle and rapid urbanization [5]. Newly acquired purchasing power has facilitated the adoption of emerging technologies such as smart phones and electronic toys [5]. These devices have contributed tremendously to sedentary behavior, low physical activity, and to the rising prevalence of overweight and obesity among Saudis [5].

Currently in Saudi Arabia, there is significant public health concern regarding the increases in obesity among the country’s youth. It is predicted that Saudi children that are currently overweight or obese will carry the burden of their excessive weight (and the disabilities that are associated with it) into adulthood [2, 6].

Evidence form the literature suggests that school-based interventions are suitable to address and contain the childhood obesity epidemic [7]. Identifying risk factors associated with childhood obesity and overweight would be an important step in the design and implementation of interventions that aim to reduce the burden associated with excessive body weight among vulnerable age groups in Saudi Arabia. Recognizing these risk factors among children will also aid in advising lifestyle modifications that promote healthier weights.

The objective of this study was to identify risk factors associated with being overweight or obese among school-aged children in Riyadh, Saudi Arabia.

## Methods

A case-control study was conducted in Riyadh, Saudi Arabia, from September through December of 2017. Cases were overweight or obese children and matched controls were normal weight children between the ages of 5-9 years attending school from kindergarten to fifth grade. Children and their parents were enrolled in the study to examine a broad range of factors related to being overweight or obese in childhood. The study was conducted according to the ethical standards of the institutional and national research committee. Informed consent was obtained from all parents. The study was approved by the Institutional Review Board (IRB serial number RYD-17-417780-85363; 30 of May, 2017) at the sponsoring institution.

### Sampling

A total of 492 children were enrolled in the study (246 cases and 246 controls) exceeding the required sample size of 250 participants (5% margin of error, 90% power of analysis, and minimum detectable OR of 2). Participants were recruited from 22 public schools (10 elementary schools for girls, 7 elementary schools for boys, and 5 co-educational kindergartens). Schools were systematically chosen based on first stratifying the city of Riyadh in to regions (north, south, central, east, and west), then randomly selecting schools from each stratum after obtaining a listing of all schools in each region and the number of enrolled students in each school. Two schools for girls, two schools for boys from the southern and northern regions, and one school from the eastern and western regions (one school was selected because boys’ schools in the two regions were larger than in other regions with regard to the number of students), and one co-educational kindergarten were chosen for the purpose of this study. Coded questionnaires and consent forms were sent to the parents and all parents who signed the consent forms and returned the filled questionnaire to the school administration within 2-3 days were enrolled in the study. Any children whose parents reported a major health condition that may act as a confounding factor (such as heart disease or diabetes) or any type of disability were excluded from the study sample. We attempted to recruit between 11 and 15 cases from each school, matched with the same number of controls from the same school, grade level, age, and gender. The school faculty made initial selection of 11 to 15 cases by visually assessing whether they were children with overweight/obesity and then selected controls who visually appeared to be normal weight. The standing height and weight of all the children were measured using standard procedures, their body mass index (BMI), as weight in kilograms/height in squared meter, was calculated, and they were classified as underweight, normal weight, overweight, or obese using BMI-for-age and sex growth charts according to the Centers for Disease Control and Prevention (CDC) guidelines [8]. Excluded children were replaced until the approximate sample size form each school was fulfilled. Underweight children were excluded from the sample for the purpose of this study since the aim of this case-control study is to compare cases with overweight/obesity to controls with normal weight.

### Survey development

A questionnaire developed for the purpose of the study based on previously published measures was used. The self-administered instrument contained valid and reliable scales such the Children’s Eating Behavior Inventory (CEBI) [7, 9–14]. The questionnaire collected information about parents’ demographic characteristics (level of education, employment status, monthly household income, and residence type), their self-reported weight and height for BMI calculation (weight in kilograms/ height in squared meters), and whether they had any morbidities. In addition to questions regarding child feeding practices since infancy (breastfeeding history, consumption of fruits and vegetables, consumption of fast food, soda intake, other eating behaviors, and purchase of junk food), the instrument included questions regarding the child’s daily physical activity, frequency of daily screen use (i.e., tablets, computers, smart phones, and television), and the type of behavioral reward system applied by the parents. Parental attitude regarding childhood obesity and how they perceived their child’s weight status (underweight, normal weight, overweight, or obese) were also included in the instrument. The developed questionnaire was translated into Arabic by a professional translator and reviewed by two bilingual research assistants for appropriateness of the terms used and inconsistencies. A group comprising of two nutritionists, two health educators, a pediatrician, and a public health practitioner reviewed the Arabic version of the instrument for content validity, relevance, and suitability of the different measures. Face validity was assessed by asking fifteen individuals to complete the survey and report any encountered difficulties. Questionnaire items were revised according to all recommendations and further tested among 25 parents of school-aged children, not included in the sample, for a final check of overall clarity. Test-retest reliability was also established, and 90% agreement was reported among the two administrations of the survey to the same group (*N*=30) of parents of young children within a 10-day interval between the two administrations. The group of parents who participated in test-retest reliability was not included in the study. Internal consistency of the instrument was determined as Cronbach’s alpha coefficient of 0.79.

### Data analysis

Questionnaire responses were coded, entered, and analyzed using the statistical software package STAT13 (College Station, Texas). Descriptive statistics were calculated for all study variables including demographic characteristics and socioeconomic status, parental calculated BMI, and children’s calculated BMI. Logistic regression analysis was conducted to examine the individual and combined effects of the study variables (related to feeding practices, parenting style, and perception regarding the child weight status) on the likelihood of being children with overweight/obesity versus normal weight controls. Odds ratios and 95% confidence intervals were estimated for all probable risk factors for overweight/obesity in this population. The significance level was set at *p* <0.05 for all analyses.

## Results

### Characteristics of study participants

The mean age of the study participants was 7.27 years (SD=±1.38). Of 492 respondents, 212 (43.09%) were male. Approximately one third of the sample (36.59%) reported unemployment status for the father and 76.82% of the mothers were reported as homemakers. The overall reported prevalence of overweight and obesity (according to reported weight and height) among participating parents was 82.49% (85.77% among mothers and 82.26% among fathers). Approximately half of the respondents (53.05%) were in the lower-income category with a cumulative monthly family income between 5000 Saudi Riyals (SR) and 10,000 SR.

Bivariate analysis showed that significantly greater proportion of overweight/obese children (cases) compared to normal weight children (controls) had fathers who were overweight or obese (86.99% vs. 78.87%; *p*<0.001). Also, overweight/obese children varied significantly from normal weight children according to cesarean section delivery mode (25.02% vs. 11.79%; *p*<0.001). There was a significant difference in the cases and controls regarding the fathers’ employment status; participating cases had fathers that reported being unemployed at higher proportions than controls (61.38% vs. 11.79%; *p*<0.001). Results for this section are displayed in Table 1.

**Table 1:** Characteristics of study participants

Characteristics	Total *N*=492	Cases *N*=246	Controls *N*=246	*p* value
**Child’s age in years, mean (SD)**	7.27 (1.38)	7.27 (1.38)	7.27 (1.38)	1.00
**Child’s gender**				1.00
Male	212 (43.09)	106 (43.17)	106 (43.17)	
Female	280 (56.91)	140 (56.91)	140 (56.91)	
**Number of children in the home**				0.66
1	48 (9.76)	28 (11.38)	20 (8.13)	
2-3	184 (37.40)	93 (37.80)	99 (40.24)	
≥4	260 (52.84)	125 (50.81)	135 (50.81)	
**Type of house**				0.06
Apartment	68 (13.82)	38 (15.44)	30 (12.19)	
House	424 (86.18)	208 (84.55)	216 (87.80)	
**Family income per month (SR)**				
<5000	89 (18.09)	43(17.48)	46(18.70)	
5000-10,000	261 (53.05)	120 (48.78)	141(57.32)	
>10,000 S	142 (28.86)	83 (33.74)	59 (23.98)	
**Mother’s age (*μ*=36.51; SD=5.42) (years)**				0.93
≤30	76 (15.45)	37 (15.04)	39 (15.85)	
31-40	327 (66.46)	163 (66.26)	164 (66.66)	
> 40	89 (18.09)	46 (18.70)	43 (21.54)	
**Mother’s BMI (*μ*=29.61; SD=6.31)**				0.095
Normal weight	85 (17.30)	35 (14.23)	50 (20.32)	
Overweight/obese	407 (82.72)	211 (85.77)	196 (79.67)	
**Mother’s education**				0.92
Middle school or less	183 (37.19)	92 (37.40)	91 (36.99)	
High school	146 (29.67)	71 (28.86)	75 (30.49)	
University and above	163 (33.13)	83 (33.74)	80 (32.52)	
**Mother’s employment status**				0.05
Employed	114 (23.17)	66 (26.83)	48 (19.51)	
Homemaker	378 (76.82)	180 (73.17)	198 (40.24)	
**Type of child delivery**				<0.001
Vaginal	401 (81.50)	182 (73.98)	217 (88.21)	
Cesarean	91 (18.50)	62 (25.20)	29 (11.79)	
**Gestational diabetes**				0.44
Yes	70 (14.23)	38 (15.45)	32 (13.01)	
No	422 (85.77)	208 (84.55)	214 (86.99)	
**Father’s age (*μ*=42.28; SD=6.06) (years)**				0.28
≤30	17 (3.46)	10 (4.06)	7 (2.85)	
31-40	202 (41.06)	108 (43.90)	94 (38.21)	
> 40	273 (55.49)	128 (52.03)	145 (58.94)	
**Father’s BMI (*μ*=29.40; SD=6.66)**				0.029
Normal weight	82 (16.73)	32 (13.00)	50 (20.32)	
Overweight/obesity	408 (82.92)	214 (86.99)	196 (78.86)	
**Father’s education**				0.72
Middle school or less	84 (17.07)	44 (17.89)	40 (16.26)	
High School	265 (53.86)	128 (52.03)	137 (55.69)	
University and above	143 (29.07)	74 (30.08)	69 (28.05)	
**Father’s employment status**				<0.001
Employed	312 (65.41)	95 (38.62)	217 (88.21)	
Non-employed	180 (36.59)	151 (61.38)	29 (11.79)	

### Child’s feeding practices, parenting style, and perception regarding child weight status

The percentage children who were frequent consumers of snacks was significantly greater among the overweight/obese cases as opposed to normal weight controls (62.60% vs. 41.46%; *p*<0.001). Also, significantly greater proportion of frequent users (>2 h per day) of screen time (television, computer, tablet, and phone) was among the cases with overweight/obesity compared to normal weight children (43.90% vs. 32.32%; *p*=0.009). Children who spent 30 min or more in active play on a daily basis were more likely to be normal weight controls than overweight/obese cases (34.55% vs. 19.51%, *p*<0.001). Additionally, there was a significant difference between the cases and controls regarding their parental perception of their body weight with the highest percentage of incorrect perceptions being among the children who were overweight or obese (51.22% vs. 29.27%; *p*<0.001). Breastfeeding the child during infancy, consumption of sugary drinks, consumption of fruits and vegetables, eating breakfast regularly, eating fast food, and rewarding the child with sugary treats did not significantly vary between cases and controls. Results for this section are displayed in Table 2.

**Table 2:** Parental reported child’s feeding practices, parenting style, and perceptions regarding child weight status

Variable	Total ***N***=492	Cases ***N***=246	Controls ***N***=246	***p*** value
**Feeding practices**				
Breastfeeding duration (months)				0.26
None	88 (17.89)	51(20.73)	37 (15.04)	
≤ 6	226 (45.93)	109 (44.31)	117 (47.56)	
>6	178 (72.36)	86 (34.96)	92 (37.40)	
Frequent snacking				<0.001
Yes	236 (47.97)	144 (58.54)	92 (18.70)	
No	256 (52.03)	102 (31.30)	154 (41.46)	
Consumption of sugary drinks				0.93
Yes	271 (55.08)	136 (55.28)	135 (54.88)	
No	221 (44.92)	110 (44.71)	111 (45.12)	
Consumption of vegetables/fruits				0.97
Yes	432 (87.80)	215 (87.40)	217 (88.21)	
No	60 (12.19)	31 (12.60)	29 (11.79)	
Eating breakfast regularly				
Yes	454 (92.28)	227 (92.28)	227 (92.28)	1.00
No	38 (15.45)	19 (7.72)	19 (7.72)	
Eating fast food				0.50
≤ 1 time/week	337 (68.50)	165 (67.07)	172 (69.92)	
>1 time/week	`155 (31.50)	81 (32.93)	74 (30.08)	
**Parenting style**				
Child is rewarded with candy/sweets				0.07
Yes	129 (26.22)	67 (27.23)	62 (25.20)	
No	363 (73.78)	179 (72.76)	184 (74.80)	
Child allowed to buy junk food				0.06
Yes	62 (12.60)	24 (9.76)	38 (15.45)	
No	430 (87.39)	222 (90.24)	208 (42.28)	
Child’s allowed screen time^a^				0.009
≤2 h/day	304 (61.79)	138 (56.10)	166 (67.48)	
>2 h/day	188 (38.21)	108 (43.90)	80 (32.52)	
Child’s daily time in active play^b^				<0.001
<30 min	359 (72.97)	198 (80.49)	161 (65.44)	
≥30 min	133 (27.03)	48 (19.51)	85 (34.55)	
**Parental perception of child’s weight**^c^				<0.001
Correct	294 (59.75)	120 (48.78)	174 (70.73)	
Incorrect	198 (40.24)	126 (51.22)	72 (29.27)	

^a^Screen time includes time spent watching television or using tablets, computers, and mobile phones outside school

^b^This question was specific for activities outside the school; including home, park, or gym activities

^c^Parental estimates of their current child weight status

### Risk factors associated with overweight and obesity

All the factors shown to be significantly associated with being overweight or obese in this group of children in bivariate analysis were entered into a multivariate logistic model to assess the independent risk factors for childhood obesity controlling for age and gender. Results for this section are displayed in Table 3. Having an unemployed father (OR=11.90; 95% CI: 7.47-18.93), a father with overweight/obesity (OR=2.04; 95% CI: 1.40-2.96), an incorrect parental perception of child’s weight status (OR=2.54; 95% CI: 1.75-3.68), cesarean delivery (OR=2.52; 95% CI: 1.56-4.09), daily time in active play for less than 30 min (OR=2.18; 95%CI: 1.44-3.28), frequent snacking (OR=1.74; 95% CI: 1.05-2.93), and screen time use for more than 2 h per day outside of school (OR=1.62; 95% CI: 1.12-2.34) were all independent risk factors for being overweight or obese among the selected cases.

**Table 3:** Results of final multivariate logistic regression analysis for factors associated with childhood overweight and obesity

Variable	Reference	OR (95% CI)	Wald	***p*** value
Cesarean delivery	Vaginal delivery	2.52 (1.56-4.09)	3.76	<0.001
Overweight/obese father	Normal weight	2.04 (1.40-2.96)	3.74	<0.001
Unemployed father	Employed	11.90 (7.47-18.93)	10.44	<0.001
Frequent snacking	Not frequent	1.75 (1.05-2.93)	2.13	0.03
Screen time for > 2 h/day	≤2 h/day	1.62 (1.12-2.34)	2.59	0.01
Daily time in active play for <30 min	≥30 min	2.18 (1.44-3.28)	3.72	<0.001
Incorrect parental perception of child weight status	Correct perception	2.54 (1.75-3.68)	4.91	<0.001

## Discussion

Due to its significant negative impact on the health and well-being of the world population, childhood obesity is a major global health issue. This study assessed a variety of risk factors contributing to childhood obesity in a sample of overweight/obese children compared with normal weight controls residing in Saudi Arabia. The findings of this study showed that paternal body mass index (BMI) is a risk factor for childhood overweight/obesity. Saudi children with an overweight/obese father were almost twice as likely to be overweight/obese compared to those with a normal weight father. These findings coincide with other studies which reported that parental BMI was a significant factor associated with childhood and teen obesity [15–17]. Previous local studies tackling childhood obesity, such as that conducted in the Al-Hasa region of Saudi Arabia, revealed that there is a higher prevalence of obesity and overweight among children whose parents are also overweight/obese [18]. In comparison to the study conducted in Al-Hasa, the current investigation was more specific as it demonstrated that father’s BMI, and not necessarily mother’s BMI, is predictive of excessive weight in children. Contrary to the findings of this study, other studies have supported the claim that maternal BMI is a more significant predictor of child weight than paternal BMI [7, 19–22]. Authors of childhood obesity literature have argued that the influence of maternal BMI can be explained by the fact that mothers are the primary caregivers of their children, and, therefore, influence the behaviors and lifestyles, such as food intake and physical activity habits, of their children [19]. It is understandable that paternal BMI would have a stronger impact on child BMI in a gender-segregated society such as Saudi Arabia in which most of the decision-making regarding household conduct and activities are the responsibility of the father [14]. Our findings showed the prevalence of overweight or obesity among fathers to be alarmingly high in the sample analyzed. Regarding eating behavior, children in the sample who were frequent snack eaters were significantly more likely to be overweight or obese compared to those who did not snack. These findings highlight the importance of planning and implementing awareness campaigns that are targeted toward parents and which place special emphasis on dietary behavior and how it affects weight gain and lifetime health. Results from a national survey showed that obese children between the ages of 2 and 5 years are at greater risk of becoming obese adults [17]. Paternal BMI and eating behavior are modifiable risk factors for childhood obesity in Saudi Arabia. These risk factors can be addressed with public health efforts and through the implementation of planned and targeted interventions.

Daily screen use was identified as a modifiable risk factor for overweight and obesity in this study. Using screens for more than 2 h per day increased the risk of being overweight/obese among this group of children. Screen time has been associated with increased risk of being overweight or obese among children in the literature [23, 24]. Children who spend long hours using screens tend to snack while doing so, and eventually, gain weight because they increase their energy intake while seated for a long period of time. Additionally, excessive media exposure has been repeatedly linked to unhealthy life choices [25, 26]. It is worth noting that significant time spent watching television or playing video games was associated with physical inactivity, which in turn is a major risk factor for being overweight or obese in all age group [2, 20, 21, 27, 28]. Children in our study who spent 30 min or more in active play on a daily basis were almost half as likely to be overweight or obese. It is important that parents abide by the recommendations for screen time use among children and employ strict parenting when it comes to the excessive use of screens [29].Controlling screen time use among children and encouraging active play is challenging in Riyadh, Saudi Arabia, due to limited access to neighborhood parks that are safe for children, and unfavorable weather conditions with excessive heat throughout most of the year. Children in Riyadh seek entertainment with electronic devices because they spend long hours indoors with their caregivers, and in private cars commuting to schools in a country with a male dependent driver system that lacks public transportation [30]. Recently, women were permitted to drive in Saudi Arabia. However, the practice of driving remains limited to very few women with jobs and from families that perceive it as culturally acceptable. Public health efforts should focus on planning for accessible indoor exercise facilities and on finding strategies that parents can apply in order to replace the frequent use of screen time.

Additionally, findings from this study suggested cesarean section delivery as risk factor for childhood obesity. This finding is consistent with a prospective cohort study in the literature that reported a fivefold higher odds of childhood obesity associated with cesarean delivery [31, 32]. Cesarean delivery is a non-modifiable risk factor for childhood obesity in this population. However, the deployment of a health message informs that cesarean delivery may increase the likelihood of childhood obesity to parents that are electively considering cesarean delivery, may be beneficial.

Although not all indicators in this study had a significant association with childhood obesity, some family characteristics, such as father unemployment status was shown to be a factor. Unemployment could be a proxy for low family income. This finding coincides with a previously conducted study linking a low socioeconomic status with childhood obesity [33, 34]. This new finding is alarming and should be considered by policy makers as they strategically plan to improve the country’s health and economy.

Parental perception about child weight status was significantly associated with child weight. These results are in accordance with findings from other studies [7]. A meta-analysis suggested that almost 50% of all the parents underestimate their children’s weight status [35, 36]. This proportion was increased among parents of young children (aged 2–6 years) [35, 37]. It has been previously documented that precise parental recognition of the weight of their children is related to a willingness to make changes associated with weight improvement [35, 36].

Obesity among children is a serious global health concern and has profound negative implications that can extend into adulthood. Our findings indicate that the main risk factors contributing to childhood obesity among Saudi Arabian children in Riyadh include paternal BMI, paternal unemployment, dietary behavior in the form of frequent snacking, screen time exceeding 2 h per day, less than 30 min of daily active play, cesarean delivery, and incorrect parental perceptions of child weight status. The evidence from this study reinforces the importance of educating and raising awareness about childhood obesity. This education should be implemented within families, schools, communities, and among policy makers.

Change is required at a multitude of levels. Policy makers should support efforts for the implementation and regulation of interventions aimed at reducing childhood obesity. Further research is needed at the national level to better address the risk factors associated with obesity among children that were identified through this study.

This study includes limitations. The first major limitation was that parental data (such as age, height, and weight) was self-reported rather than measured by research assistants or taken from medical files. This may have resulted in reporting error as under or over estimations may have occurred. In addition, some study variables were not measured objectively. This study relied on parents to recall such things as their children’s level of physical activity, meal duration, fast food meal frequency. This may have resulted in some recall error. Our study had a 15.3% non-respond rate.

## Data Availability

The datasets analyzed during the study are available from the corresponding author on reasonable request and after approval of the concerned authorities in the organization.

## Abbreviations

CI: Confidence interval
OR: Odds ratio
BMI: Body mass index
CEBI: Child eating behavior inventory
IRB: Institutional review board
